# Association of Previous Measles Infection With Markers of Acute Infectious Disease Among 9- to 59-Month-Old Children in the Democratic Republic of the Congo

**DOI:** 10.1093/jpids/piy099

**Published:** 2018-10-19

**Authors:** Hayley R Ashbaugh, James D Cherry, Nicole A Hoff, Reena H Doshi, Vivian H Alfonso, Adva Gadoth, Patrick Mukadi, Stephen G Higgins, Roger Budd, Christina Randall, Emile Okitolonda-Wemakoy, Jean Jacques Muyembe-Tamfum, Sue K Gerber, Anne W Rimoin

**Affiliations:** 1 Department of Epidemiology, Fielding School of Public Health, University of California, Los Angeles; 2 David Geffen School of Medicine, University of California, Los Angeles; 3 School of Medicine, Kinshasa University, Democratic Republic of the Congo; 4 OpGen Incorporated, Gaithersburg, Maryland; 5 Dynex Technologies Incorporated, Chantilly, Virginia; 6 Kinshasa School of Public Health, Democratic Republic of the Congo; 7 National Institute for Biomedical Research, Kinshasa, Democratic Republic of the Congo; 8 Bill and Melinda Gates Foundation, Seattle, Washington

**Keywords:** cough, diarrhea, fever, immunosuppression, measles

## Abstract

**Background:**

Transient immunosuppression and increased susceptibility to other infections after measles infection is well known, but recent studies have suggested the occurrence of an “immune amnesia” that could have long-term immunosuppressive effects.

**Methods:**

We examined the association between past measles infection and acute episodes of fever, cough, and diarrhea among 2350 children aged 9 to 59 months whose mothers were selected for interview in the 2013–2014 Democratic Republic of the Congo (DRC) Demographic and Health Survey (DHS). Classification of children who had had measles was completed using maternal recall and measles immunoglobulin G serostatus obtained via dried-blood-spot analysis with a multiplex immunoassay. The association with time since measles infection and fever, cough, and diarrhea outcomes was also examined.

**Results:**

The odds of fever in the previous 2 weeks were 1.80 (95% confidence interval [CI], 1.25–2.60) among children for whom measles was reported compared to children with no history of measles. Measles vaccination demonstrated a protective association against selected clinical markers of acute infectious diseases.

**Conclusion:**

Our results suggest that measles might have a long-term effect on selected clinical markers of acute infectious diseases among children aged 9 to 59 months in the DRC. These findings support the immune-amnesia hypothesis suggested by others and underscore the need for continued evaluation and improvement of the DRC’s measles vaccination program.

## Teaser

In this study, we assessed the association of previous measles infection with episodes of fever, cough, or diarrhea in a low-vaccine-coverage resource-poor area, and our results suggest that measles infection might have a long-term effect on markers of acute infectious disease in our study population.

Measles is primarily a highly transmissible childhood viral disease caused by a single-stranded RNA paramyxovirus (genus *Morbillivirus*). Vaccination with currently used measles vaccines results in long-lasting immunity; seroconversion rates are 85% when children are vaccinated at 9 months of age and approximately 95% after 1 dose of vaccine among children aged 12 months or older [1, 2]. World Health Organization guidance recommends that all countries include a second routine dose of measles vaccine, even in countries with low national coverage of the first dose [3]. Countries in which measles is endemic, such as the Democratic Republic of the Congo (DRC), should routinely vaccinate children at 9 months and then at 12 to 15 months of age to protect those who might not have developed immunity after their first dose [4]. In 2015, the DRC reported 79% national coverage for the first dose of measles vaccination [3], and measles ranked 15th among the top causes of death in children less than 5 years of age in the DRC in 2016 [5].

In addition to its high burden in DRC children, measles has more severe effects on child health as a result of widespread immunosuppression that stems from malnutrition. The DRC’s 2013–2014 Demographic and Health Survey (DHS) found that 23% of children younger than 5 years were acutely malnourished (wasted), and 43% were chronically malnourished (stunted) [6]. Malnutrition has been linked to dysfunction of cell-mediated immunity [7] and, in poor regions, increased risk of morbidity and death in those with measles. In 1998, Belamarich [8] estimated a 5% to 25% measles mortality rate in developing areas; for comparison, a 1% rate was noted in the United States during the measles epidemic in 1989–1990. In addition, measles infection greatly increases the risk of corneal ulceration and subsequent blindness among African children, particularly in those who are undernourished [1, 9].

Even in healthy children, measles virus infection is associated with transient but severe immunosuppression [10, 11]. This infection-associated lymphopenia is short lived (approximately 1 week in duration), and only 1% to 5% of total peripheral lymphocytes are infected [10, 12, 13]. However, it has long been known that measles virus can grow in the spleen, lymph nodes, and tonsils [14], and studies finding high percentages of measles virus–infected cells in lymphoid tissue [12] led de Vries et al [11] to reconsider lymphocyte depletion as a possible mechanism for virus-induced immunosuppression. They suggest that the marked increase of measles virus–specific and bystander lymphocytes in response to infection resolves measles’ acute global lymphopenia yet masks subsets of immune-mediated lymphocyte depletion. In addition, in a large population study in England, Wales, the United States, and Denmark, in which mortality rates in the pre–measles vaccine era were compared with those in the vaccine era, the authors noted that the increased number of deaths from nonmeasles infectious diseases, hypothesized to be a consequence of prolonged immunosuppression that resulted from a depletion of memory lymphocytes, after high population levels of measles was not transient but, rather, lasted 2 to 3 years [15].

In a resource-poor area such as the DRC, limited health care services, poor nutrition, and high levels of infectious disease make host immune function of crucial public health importance. Therefore, identifying risk factors (such as previous measles infection) for adverse disease outcomes (such as fever, cough, or diarrhea) that result from postmeasles immunosuppression can result in valuable recommendations for DRC public health policy and practice.

## METHODS

### Data Source and Study Population

The 2013–2014 DHS was conducted between November 2013 and February 2014 and is a nationally representative survey based on a stratified 2-stage cluster design; the first stage consisted of enumeration-area formation, and the second stage involved sampling households from each enumeration area [16–18]. In the first stage, a stratified sample of geographic locations, or clusters (n = 540), was selected with proportional probability according to size. Complete listings of households within each cluster were created and selected with equal probability (n = 9000). For this study, 18827 women aged 15 to 49 years and 8656 men aged 15 to 59 years in 50% of the selected households were interviewed.

The DHS survey collected biomarker data on children if they were 6 to 59 months of age and in a household in which a male interview was conducted. Collected data included weight, anemia status, health outcomes, vaccination history, and vaccine-preventable disease serology. The University of California, Los Angeles (UCLA)–DRC laboratory at the National Laboratory for Biomedical Research in Kinshasa processed dried blood spots (DBSs) collected from participants after parental consent and assessed them for immunity to vaccine-preventable diseases. Survey data were recorded on paper questionnaires and transferred to an electronic format using the Census and Survey Processing System (US Census Bureau, ICF Macro). Data were entered twice, and the resulting 2 data sets compared and verified.

### Laboratory Analysis

DBS samples were extracted using a modified extraction protocol [19] and processed at the UCLA-DRC laboratory at the National Laboratory for Biomedical Research in Kinshasa. The DBS extraction and assay protocol and multiplex technology have been described elsewhere [20]. The Dynex Multiplier chemiluminescent automated immunoassay platform with a research-use-only SmartPLEX assay for measles, mumps, rubella, varicella-zoster virus, and tetanus (Dynex Technologies, Chantilly, Virginia) was used to test samples for immunoglobulin G antibody response. An assay score (AS) was calculated for each assay as the ratio to a five-donor, pooled positive control included in each run. The limit of quantitation (LOQ) was calculated as an AS of 0.03, and this value (as a supplement to maternal report of measles) was used to indicate previous infection among children for whom a history of measles was reported. We used the LOQ in conjunction with maternal report to avoid missing cases of infants who might have had measles but failed to mount a robust immune response because of maternal antibody interference. As an internal validation, we compared measles antibody titers of children with a maternal measles report and those in children with no report, and we also conducted this analysis while limiting reports to those from mothers with a higher level of education to examine potential differences in reporting according to maternal education.

### Study Design

We assessed the association of past measles disease with an increased prevalence of infectious disease markers among 2350 children aged 9 to 59 months. Mothers selected for the DHS household interview reported whether their child had ever had measles and his or her approximate age (in months) at the time of illness. Of these children, those whose AS was less than the minimum antibody level of 0.03 were considered to be uninfected. This minimum antibody level was used to indicate measles infection among those children for whom measles had already been reported, whereas antibody found among those for whom no disease was reported was considered vaccine induced. A child was considered vaccinated if, during the interview, his or her mother presented the interviewer with a vaccination card (provided by a healthcare worker) that contained the date that the child was vaccinated against measles [17]. Unvaccinated children were those reported as such in the DHS survey. Because of the cross-sectional nature of this study, we assessed the prevalence of maternally reported acute infectious disease markers (fever, cough, and diarrhea) during the 2-week reporting period before interview, as defined in the DHS survey. The final sample size was determined by variables available for each child.

### Statistical Analysis

DHS surveys oversample or undersample different geographic areas, and the inclusion of individual-, stratum-, and cluster-level weights is required for unbiased estimates and confidence intervals (CIs) [18]. We used methods in SAS 9.4 (SAS Institute, Cary, North Carolina) that accounted for complex survey design [21], and to estimate variance correctly, all final analyses were single level.

In addition to a descriptive analysis according to measles status, we performed a second descriptive analysis according to the distribution of fever, cough, diarrhea, and fever/cough/diarrhea (all 3 reported within the previous 2 weeks) outcomes to assess the imbalance of fever, cough, and diarrhea outcomes across demographic and socioeconomic variables. A logistic regression model that accounted for survey design was used to examine the association between measles and episodes of fever, cough, or diarrhea. The model was run again to examine the association of measles with the occurrence of all 3 outcomes within the same 2-week period and then to examine the association of outcomes with time (months) since measles. To account for potential miscategorization of time since measles infection, we categorized this analysis in 2 ways, by dividing at <12, <24, and 24 to 58 months (model 1) and by dividing by approximately 9-month intervals up until 36 months and limiting the recall period to 3 years or less (model 2). To examine trends beyond the expected window for short-term immunosuppression caused by measles, children for whom measles infection was reported to have occurred less than 2 months before the interview were removed from the time-since-measles analysis. Because of the small cell sizes in this analysis, we also show the frequency data for all outcomes of interest within each month category.

### Sensitivity Analysis

To examine potential effects of misclassification on odds ratio estimates, we included a sensitivity analysis in which we selected a simple random sample of measles cases to be considered misclassified and included these selected cases in the nonmeasles group. In their study, Doshi et al [22] found that 48.6% of measles-like illness reports made using the DRC’s case-based surveillance system in 2010–2012 were laboratory confirmed to be positive. We therefore ran 4 models, first considering only 29% of cases to be true positive and the remaining 71% to be false positive (and thus categorized as nonmeasles), and then we did the same for 39%, 49%, and 59% of the 193 measles cases in the full sample.

### Covariate Selection

Covariate selection was based on a priori confounders (age, sex) and other potential confounders identified in the literature. Because areas with a high level of malnutrition tend to manifest greater severity in measles cases, we controlled for chronic malnutrition by categorizing children as stunted or normally or overnourished, as calculated in the DHS and according to National Center for Health Statistics/Centers for Disease Control and Prevention/World Health Organization international reference standards [17]. Malaria status was included because it is a common cause of febrile illness [23] in this region, and vaccination, breastfeeding, maternal education (7 or more years), and low parity were included because they have been found to be protective against child death [24–26]. Hobcraft et al [26] also found that 3 or more births in a 2- to 6-year period potentially increases the mortality rate in children younger than 3 years, and we applied this criterion to define high versus low parity. To control for poverty, we created a binary variable from the DHS categorical wealth index variable (in quintiles) by categorizing the 2 lowest categories (“poorest” and “poorer”) as “poor” and the 3 highest categories (“middle,” “wealthy,” and “wealthiest”) as “middle income/wealthy.” We previously found that measles vaccination and wealth index have an interactive effect on markers of infectious disease outcomes and thus included this interaction in our analysis; likewise, measles vaccination and DRC province have an interactive effect that we incorporated into our final model for fever, cough, and diarrhea outcomes. In addition, because the DHS wealth index variable depends on items more frequently found in urban than in rural residences, we included an interaction variable (wealth index × residence) in the final model [27].

Although measles is known to have seasonal variation in nonindustrialized nations [28], the inclusion of seasonality was found to be minimally informative in this analysis and was not included in the final model because the data were collected within a short 4-month period and weather patterns across the DRC differ.

Although previous work on long-term immune effects of measles examined death as an outcome [15], data on all-cause deaths between measles and nonmeasles cases were not available. Because the DHS primarily provides information on living children, we examined clinical markers of infection as our outcome of interest, which, to our knowledge, has not yet been examined in this context. Analyses were completed using SAS 9.4.

Ethical approval was obtained by the UCLA Fielding School of Public Health, the Kinshasa School of Public Health, and the Centers for Disease Control and Prevention. Because the children were younger than the standard age of assent, the parent or guardian of each participating child provided consent on that child’s behalf.

## RESULTS

### Internal Validation of Measles Categorization

Among the children with a maternal report of measles, 193 (77%) of 252 had a measles antibody level of at least 0.03 AS. As an internal validation, we examined the correlation of measles antibody titer with maternal report of measles infection. Median measles antibody titer levels were higher among children with a maternal report of measles infection (median AS value, 0.507 [95% CI, 0.223–0.791]) than among those with no report (median AS value, 0.176 [95% CI, 0.152–0.199]) (Supplementary Table 1). To explore the potential effect of maternal education on measles reporting, we conducted this analysis limited to mothers with 7 or more years of education and again found that children with maternal report of measles infection had higher measles antibody titer levels (median AS value, 0.742 [95% CI, 0.009–1.476]) than those with no report of measles infection (median AS value, 0.203 [95% CI, 0.160–0.242]), although the CI for the group with report of measles infection among more highly educated mothers (n = 39) is notably wider than that from the group that included all observations (n = 705).

### Descriptive Analyses

We observed differences (Table 1) between history of measles disease and vaccination status (*P* = .0036, Wald χ^2^), age (*P* < .0001, Wald χ^2^), breastfeeding status (*P* < .0001, Wald χ^2^), years of maternal education (*P* = .0086, Wald χ^2^), DRC province (*P* = .0006, Wald χ^2^), and fever outcome (*P* = .0485, Wald χ^2^). Examination of demographic and socioeconomic variables according to fever, cough, and diarrhea outcomes (Supplementary Table 2) revealed differences according to vaccination status for diarrhea outcome (*P* = .0131, Wald χ^2^), age (*P* = .0025, .0032, <.0001, and .0003 for fever, cough, diarrhea, and fever/cough/diarrhea, respectively, Wald χ^2^), and breastfeeding status for all outcomes except cough. Other differences included cough outcome according to wealth index and residence (*P* = .0114, Wald χ^2^) and fever outcome according to malaria result and measles history (*P* = .0018, Wald χ^2^). Controlled for covariates, logistic regression analyses (Table 2) revealed that children with a history of measles disease had greater odds of experiencing a fever episode within the 2 weeks before interview (odds ratio [OR], 1.80 [95% CI, 1.25–2.60]). Measles vaccination was associated with decreased odds of fever, diarrhea, and fever/cough/diarrhea, and a positive malaria test result was associated with increased odds of fever. The sensitivity analysis that examined changes in outcome according to potential percentage of measles cases misclassified (Supplementary Table 3) revealed a continued association with previous measles infection and increased odds of fever if as many as half of the measles reports had been misclassified. The trend in association continued for higher levels of misclassification but was not statistically significant.

**Table 1.** Descriptive Data of Children Aged 9 to 59 Months With and Those Without a History of Measles Infection

	All Children		Children With Measles Infection History		
Variable	n	% of Total	n	% of Category	P^a^
Vaccinated against measles					**.0036**
No	1608	68	157	10	
Yes	742	32	36	5	
Age (mo)					**<.0001**
9–11	264	11	11	4	
12–23	725	31	36	5	
24–35	547	23	42	8	
36–47	434	18	57	13	
48–59	381	16	47	12	
Breastfeeding					**<.0001**
Never	52	2	4	8	
Past	1395	59	151	11	
Current	964	41	38	4	
Maternal education					**.0086**
<7 years	1605	68	154	10	
≥7 years	745	32	39	5	
DRC province					**.0006**
Kinshasa	149	6	8	5	
Bandundu	318	14	20	6	
Bas-Congo	83	4	0	0	
Equateur	366	16	42	11	
Kasai-Occidental	234	10	6	3	
Kasai-Oriental	287	12	35	12	
Katanga	282	12	23	8	
Maniema	87	4	5	6	
Nord-Kivu	245	10	19	8	
Orientale	165	7	31	19	
Sud-Kivu	133	6	4	3	
Sex					.5963
Male	1134	48	97	9	
Female	1216	52	96	8	
Wealth index^b^					.3213
Poor	1149	49	104	9	
Middle income/wealthy	1201	51	89	7	
Residence					.2117
Urban	704	30	48	7	
Rural	1646	70	145	9	
Chronically malnourished^c^					.2924
Yes	1122	48	99	9	
No	1228	52	94	8	
Children <5 years old in household					.5027
<3 children	1602	68	126	8	
≥3 children	747	32	67	9	
Malaria (blood smear) result					.4685
Negative	1825	78	145	8	
Positive	525	22	48	9	
Fever					**.0485**
Yes	830	35	84	10	
No	1519	65	109	7	
Cough					.5885
Yes	832	35	73	9	
No	1518	65	120	8	
Diarrhea					.7523
Yes	534	23	42	8	
No	1816	77	151	8	
Fever/cough/diarrhea					.3323
Yes	200	9	21	11	
No	2150	91	172	8	
Total	2350	—	193	8	

^a^Wald χ^2^ test for independence of measles status and row variables. Values in bold type indicate statistical significance.

^b^Wealth index is the Demographic and Health Survey composite measure of a household’s cumulative living standard. On the basis of household ownership of previously selected assets and using principal components analysis, households were placed within 1 of 5 quintiles. For the dichotomized variable, we combined the 2 lowest categories into the “poor” category and the 3 wealthiest into the “middle income/wealthy” category.

^c^Calculated according to the National Center for Health Statistics/Centers for Disease Control and Prevention/World Health Organization international reference standard for height and age, dichotomized as −2.0 to less than or equal to −3.0 standard deviations (SDs) below the mean for chronically malnourished children and normal to ≥3.0 SDs above the mean for normally and overnourished children.

**Table 2.** Association of Measles Disease History With Acute Infectious Disease Episodes of Fever, Cough, Diarrhea, and Fever/Cough/Diarrhea in the 2 Weeks Before Interview Among Children Aged 9 to 59 Months

Variable	OR (95% CI) for^a^:			
	Fever^b^	Cough	Diarrhea	Fever/Cough/Diarrhea^c^
Measles^d^	**1.80 (1.25–2.60**)	1.24 (0.82–1.86)	1.24 (0.80–1.93)	1.74 (0.96–3.15)
Selected covariates				
Received measles vaccination	**0.53 (0.35–0.82**)	0.76 (0.51–1.13)	**0.25 (0.17–0.37**)	**0.51 (0.30–0.88**)
Malaria positive	**1.54 (1.16–2.03**)	0.89 (0.66–1.19)	1.03 (0.78–1.35)	0.94 (0.61–1.45)

Abbreviations: CI, confidence interval; OR, odds ratio.

^a^Values in bold type indicate statistical significance.

^b^Controlled for the following additional covariates: measles vaccination (vx), wealth index (WI), vx × WI interaction, breastfeeding, maternal education, parity, age, sex, malaria-positive status, rural versus urban residence, residence × WI interaction, (old) DRC province, vx × DRC province interaction, and chronic malnutrition (according to National Center for Health Statistics/Centers for Disease Control and Prevention/World Health Organization international reference standards for height and age standard deviations).

^c^Because of the reduced number of outcomes for fever/cough/diarrhea, the vx × DRC province interaction variable was removed from the model.

^d^We used 2350 observations in the regression model for all outcomes of fever, cough, diarrhea, and fever/cough/diarrhea.

### Time-Since-Measles Analysis

Of 177 children for whom the date of measles infection was given and who met inclusion criteria, the median time since disease was 14.8 months (standard error, 1.08 months; range, 0–57 months). We performed logistic regression analyses to examine associations with acute fever, cough, or diarrhea episodes and a categorical “time (months) since measles” variable (Table 3). Trends of increased odds for fever outcome among children with measles disease were found in all 3 time categories (reference category, no history of measles) but were statistically significant in only the 13–24 month category (OR, 2.08 [95% CI, 0.96–4.51]) for model 1 and for the 10- to 18-month (OR, 1.93 [95% CI, 1.03–3.62]) and 19- to 27-month (OR, 3.01 [95% CI, 1.18–7.67]) age categories for model 2. Although not all estimates were statistically significant, the fever outcome showed consistently elevated trends. In Supplementary Table 4, model 1 data show that approximately 50% of the children with measles disease history in the previous 2 years also had a report of fever within the previous 2 weeks, compared with 35% of the children with no measles disease history and a report of recent fever. Model 2, which categorized time in 8- to 9-month intervals, shows a similar trend for children with history of measles disease in the previous 3 years.

**Table 3.** Association of Time in Months Since Measles Disease With Acute Infectious Disease Episode of Fever, Cough, or Diarrhea in the Previous Two Weeks Among Children 9–59 Months of Age.

Time Since Measles (months)^b^	Fever	Cough	Diarrhea
	OR and 95% CI^a^	OR and 95% CI	OR and 95% CI
Model 1 (2–57 months)			
2–12 (n = 62)	2.08 (0.96–4.51)	1.53 (0.70–3.33)	1.02 (0.50–2.11)
13–24 (n = 54)	**2.14 (1.12–4.08**)	0.94 (0.41–2.17)	1.44 (0.75–2.78)
25–57 (n = 60)	1.54 (0.82–2.87)	1.37 (0.76–2.46)	1.58 (0.74–3.37)
Model 2 (2–36 months)			
2–9 (n = 54)	1.79 (0.80–3.99)	1.71 (0.74–3.97)	1.14 (0.50–2.63)
10–18 (n = 46)	**1.93 (1.03–3.62**)	1.02 (0.50–2.10)	1.36 (0.68–2.71)
19–27 (n = 24)	**3.01 (1.18–7.67**)	1.54 (0.63–3.75)	1.08 (0.35–3.32)
28–36 (n = 30)	1.94 (0.80–4.71)	1.59 (0.68–3.73)	2.58 (0.93–7.13)

^a^Controlling for the following additional covariates: measles vaccination (vx), wealth index (WI), vx*WI interaction, breastfeeding, maternal education, parity, age, sex, malaria positive status, rural versus urban residence, residence*WI interaction, (old) province, vx*province interaction, and chronic malnutrition (according to NCHS/CDC/WHO international references standard for height/age SD).

^b^Reference = No history of measles (n=2,157).

## DISCUSSION

The results of our analysis suggest that previous measles infection, as reported via maternal recall and meeting serologic criteria, was associated with increased odds of fever outcomes among children aged 9 to 59 months, which supports the hypothesis of immune-amnesia leading to a prolonged period of increased risk for death as a result of non-measles infectious diseases. The association of infectious disease markers with time in months since measles infection also suggests some support for this hypothesis. Although most estimates were not statistically significant, this lack of statistical significance might have been a result of the small number of children with a history of measles in each category, and we found a trend of prolonged association with fever beyond the weeks after measles infection. The lack of association between measles and cough might have been a result of environmental or indoor pollution having a greater effect on respiratory illness than previous history of measles disease since environmental pollution is known to cause respiratory symptoms in children [29], and indoor air-pollution exposure is a public health concern in many areas of sub-Saharan Africa [30].We also found no association between past measles infection and recent diarrhea. A possible reason is that measles virus might not target all intestinal lymphoid tissue. Although findings of de Swart and coworkers [12] indicated that lymphoid tissue in the small and large intestinal submucosa of the macaque is a target for measles virus, no evidence of infected cells in the epithelial layer was found. If prolonged measles-induced immunosuppression were to affect primarily cells of the submucosa and leave lymphoid cells in the intestinal epithelium unaffected, this may suggest that these intact cells could provide some compensation for immunomodulation caused by submucosal lymphoid cell depletion.

We found that measles vaccination had a protective association against fever, diarrhea, and fever/cough/diarrhea. Measles vaccination may exert beneficial nonspecific effects that are thought to affect resistance to infectious diseases other than the targeted disease [31], and both observational and randomized trials from low-income countries have found an association between measles vaccination and reduced overall death and morbidity that cannot be explained by the prevention of measles alone [32–39]. A potential reason for why a protective association between measles vaccine between all 3 symptoms, but not cough alone, was found is that, as described above, other risk factors may mask a potential underlying association between measles vaccination and cough that becomes statistically significant when a child experiences all 3 outcomes. Alternatively, children who experience all 3 symptoms might be more likely to have a cough of infectious origin than those children who had only cough. Malaria symptoms and treatment are commonly associated with fever [23], which agrees with the associations found in our analyses.

Additional research is warranted to determine how much of the association of protection against fever, diarrhea, and fever/cough/diarrhea is a result of measles vaccination itself apart from the prevention of measles disease. The nearly null association with cough could be because of the high prevalence of asthma and other chronic respiratory disorders that are significant causes of morbidity and death in sub-Saharan Africa [40], but that we were unable to assess it in this study.

To our knowledge, ours is the first study to have examined the association of previous measles infection with a prolonged increase in the prevalence of acute infectious disease clinical markers. The strengths of this study include its large, nationally representative sample, increased confidence in maternal measles report because of serologic test results that revealed differences in median serum antibody levels between children with and those without a report of measles infection, and collection of the date of measles infection to assess its association with episodes of fever, cough, or diarrhea over time. There were several limitations to this study. (1) Measles reports were not laboratory confirmed. Although measles diagnosis based on clinical signs has been shown to be unreliable in more highly vaccinated countries, it is still thought to be a sensitive method for detecting measles infection [41, 43], and the high prevalence of measles in the DRC [42] might increase the positive predictive value (PPV) of a clinical measles diagnosis compared to that in countries with lower measles prevalence; previous work found the PPV of clinical measles diagnosis to be improved in higher-prevalence locations [43]. (2) Although clinical signs of measles, particularly Koplik spots [43], can provide a higher PPV in higher-prevalence areas, measles reports were obtained from mothers rather than trained healthcare workers and so might have been less reliable. However, previous work has indicated that maternal recall of symptoms associated with childhood deaths, including death caused by measles, in sub-Saharan Africa were reported with a high degree of accuracy, and recollections of signs and symptoms within 1 month versus within 6 months of a child’s death were found to be similar [44]. Moreover, despite the lack of healthcare practitioner reports of disease, we substantiated maternal reports of measles infection with serology and therefore lessened the possibility of exposure misclassification. If misclassification of measles did occur, it would likely be nondifferential, because inaccurate maternal memory or assessment of measles disease would likely be relatively equal across demographic groups, which would bias estimates toward the null. An exception to this tendency toward non-differential misclassification might be found among reports from more highly educated mothers. This group did demonstrate a statistically significant difference in reported measles cases compared to those from less educated mothers, but this difference could be a result of mothers in the highly educated group (in urban settings) being more likely to have vaccinated their children against measles [45]. Such differential misclassification could bias the estimate toward or away from the null [46], but we attempted to account for this limitation with a sensitivity analysis, which revealed a continued association with past measles disease and fever even if nearly half of the measles cases had been misclassified. (3) Elevated levels of malnutrition in regions such as the DRC can drive measles-related mortality rates as high as 6% [47]. Survivor bias might have occurred in our study, because the most severely affected children might have died and thus would not have been included in the analyses; however, survivor bias would likely influence estimates toward the null. (4) The cross-sectional nature of the survey might have missed acute infectious disease episodes in children who truly experienced an overall increased incidence caused by previous measles infection but who did not experience disease in the 2-week reporting window for the DHS survey.

In conclusion, our findings provide support for the hypothesis of measles-induced prolonged immunosuppression suggested by others and underscore the need for continued evaluation and improvement of the measles vaccination program in the DRC. Elevating vaccination coverage in the least-reached areas is an important component of strengthening the national vaccination program to control and eliminate measles [45]. In addition, more studies in wealthy countries could further clarify the immune-amnesia hypothesis. An increase in the number of infectious disease episodes over the long term in children who have a history of measles infection puts added strain on both the local health system and on the wellness of other children with whom the infected child comes in contact. The profound effect of measles on the lives and health of children in sub-Saharan Africa warrants concerted efforts toward the mitigation and elimination of this disease, and better defining the risks that measles poses to child health can drive and refine vaccination program policy.

## Supplementary Data

Supplementary materials are available at *Journal of the Pediatric Infectious Diseases Society* online.

piy099_suppl_Supplementary_Table_1

piy099_suppl_Supplementary_Table_2

piy099_suppl_Supplementary_Table_3

piy099_suppl_Supplementary_Table_4

## Notes

***Acknowledgments.*** We thank Jeff Gornbein and Philip Eckhoff for their thoughtful feedback on statistical analyses and interpretation and Steve Meshnik for quality control assistance.

***Disclaimer. ***The views expressed here are the authors’ alone and do not reflect the official policy or position of the Department of the Army, Department of Defense, or the US government.

***Financial support***. This work was supported by the Faucett Catalyst Fund and the Estimating Population Immunity to Poliovirus in the Democratic Republic of the Congo grant by the Bill and Melinda Gates Foundation (OPP1066684).

***Potential conflicts of interest.*** All authors: No reported conflicts of interest. All authors have submitted the ICMJE Form for Disclosure of Potential Conflicts of Interest. Conflicts that the editors consider relevant to the content of the manuscript have been disclosed.

***Previous presentation***:This work was presented at Fondation Merieux’s “Communication of vaccine benefit beyond the infection prevented” conference, Les Pensières Center for Global Health, Veyrier-du-Lac, France (4–6 December 2017); at the Society for Epidemiologic Research 2017 annual meeting in Seattle, Washington (20–23 June 2017); and at the 2016 annual American Society of Tropical Medicine and Hygiene conference, Atlanta, Georgia (13–17 November 2016).
