# Supplementary material for: Association of Previous Measles Infection With Markers of Acute Infectious Disease Among 9- to 59-Month-Old Children in the Democratic Republic of the Congo
Source: J Pediatric Infect Dis Soc. 2018 Oct 19;8(6):531–8. doi: 10.1093/jpids/piy099 (PMC6933309; doi:10.1093/jpids/piy099)
Supplement: piy099_suppl_Supplementary_Table_1 [file piy099_suppl_supplementary_table_1.docx]

| Supplementary Table 1: Median measles antibody levels in reported measles cases versus non-cases among children 9-59 months of age. | | |
| --- | --- | --- |
|  |  |  |
| **Variable** | **n** | **Median Assay Score and 95% CI** |
|  |  |  |
| **Complete dataset** |  |  |
| Measles positive | 193 | 0.507 (0.223-0.791) |
| Measles negative | 2157 | 0.176 (0.152-0.199) |
| **Limited to mothers with > 7 years of education** |  |  |
| Measles positive | 39 | 0.742 (0.009-1.476) |
| Measles negative | 705 | 0.201 (0.160-0.242) |
