# Supplementary material for: Association of Previous Measles Infection With Markers of Acute Infectious Disease Among 9- to 59-Month-Old Children in the Democratic Republic of the Congo
Source: J Pediatric Infect Dis Soc. 2018 Oct 19;8(6):531–8. doi: 10.1093/jpids/piy099 (PMC6933309; doi:10.1093/jpids/piy099)
Supplement: piy099_suppl_Supplementary_Table_2 [file piy099_suppl_supplementary_table_2.docx]

| Supplementary Table 2. Descriptive data in children 9-59 months of age with history of fever, cough, diarrhea, and fever + cough + diarrhea. | | | | | | | | | | |
| --- | --- | --- | --- | --- | --- | --- | --- | --- | --- | --- |
| **Variable** | **All children** | **(%)^1^** | **Fever** | **(%)^2^** | **Cough** | **(%)** | **Diarrhea** | **(%)** | **Fever + Cough + Diarrhea** | **(%)** |
|  |  |  |  |  |  |  |  |  |  |  |
| **Vaccinated for measles** |  |  |  |  |  |  |  |  |  |  |
| No | 1608 | 68 | 594 | 37 | 543 | 34 | 394 | 25 | 150 | 9 |
| Yes | 742 | 32 | 236 | 32 | 288 | 39 | 140 | 19 | 49 | 7 |
| **P-value^3^** |  |  | 0.0697 | | 0.1254 | | **0.0131** | | 0.0723 | |
| **Age (months)** |  |  |  |  |  |  |  |  |  |  |
| 9-11 | 264 | 11 | 102 | 39 | 112 | 42 | 84 | 32 | 32 | 12 |
| 12-23 | 725 | 31 | 291 | 40 | 285 | 39 | 244 | 34 | 82 | 11 |
| 24-35 | 547 | 23 | 196 | 36 | 198 | 36 | 120 | 22 | 49 | 9 |
| 36-47 | 434 | 18 | 135 | 31 | 123 | 28 | 46 | 11 | 24 | 6 |
| 48-59 | 381 | 16 | 106 | 28 | 114 | 30 | 40 | 10 | 13 | 3 |
| **P-value** |  |  | **0.0025** | | **0.0032** | | **< 0.0001** | | **0.0003** | |
| **Breastfeeding** |  |  |  |  |  |  |  |  |  |  |
| Never | 52 | 2 | 14 | 27 | 20 | 38 | 7 | 13 | 4 | 8 |
| Past | 1395 | 59 | 466 | 33 | 463 | 33 | 229 | 16 | 86 | 6 |
| Current | 903 | 38 | 351 | 39 | 349 | 39 | 298 | 33 | 110 | 12 |
| **P-value** |  |  | **0.0364** | | 0.1164 | | **< 0.0001** | | **0.0018** | |
| **Maternal education** |  |  |  |  |  |  |  |  |  |  |
| < 7 years | 1605 | 68 | 539 | 34 | 540 | 34 | 356 | 22 | 137 | 9 |
| ≥ 7 years | 745 | 32 | 292 | 39 | 291 | 39 | 178 | 24 | 62 | 8 |
| **P-value** |  |  | 0.1021 | | 0.0959 | | 0.4391 | | 0.9173 | |
| **Province** |  |  |  |  |  |  |  |  |  |  |
| Kinshasa | 149 | 6 | 41 | 28 | 43 | 29 | 41 | 28 | 12 | 8 |
| Bandundu | 318 | 14 | 79 | 25 | 78 | 25 | 67 | 21 | 14 | 4 |
| Bas-Congo | 83 | 4 | 31 | 37 | 20 | 24 | 15 | 18 | 2 | 2 |
| Equateur | 366 | 16 | 130 | 36 | 150 | 41 | 90 | 25 | 26 | 7 |
| Kasai-Occidental | 234 | 10 | 108 | 46 | 70 | 30 | 64 | 27 | 20 | 9 |
| Kasai-Oriental | 287 | 12 | 130 | 45 | 134 | 47 | 92 | 32 | 51 | 18 |
| Katanga | 282 | 12 | 121 | 43 | 282 | 100 | 59 | 21 | 26 | 9 |
| Maniema | 87 | 4 | 24 | 28 | 13 | 15 | 9 | 10 | 3 | 3 |
| Nord-Kivu | 245 | 10 | 51 | 21 | 91 | 37 | 26 | 11 | 10 | 4 |
| Orientale | 165 | 7 | 47 | 28 | 43 | 26 | 34 | 21 | 9 | 5 |
| Sud-Kivu | 133 | 6 | 69 | 52 | 75 | 56 | 39 | 29 | 27 | 20 |
| **P-value** |  |  | **< 0.0001** | | **0.0002** | | **0.0044** | | **0.0018** | |
| **Sex** |  |  |  |  |  |  |  |  |  |  |
| Male | 1134 | 48 | 398 | 35 | 415 | 37 | 269 | 24 | 95 | 8 |
| Female | 1216 | 52 | 433 | 36 | 417 | 34 | 266 | 22 | 105 | 9 |
| **P-value** |  |  | 0.8399 | | 0.4326 | | 0.4039 | | 0.8562 | |
| **Wealth index^4^** |  |  |  |  |  |  |  |  |  |  |
| Poor | 1149 | 49 | 402 | 35 | 361 | 31 | 272 | 24 | 100 | 9 |
| Middle income/wealthy | 1201 | 51 | 429 | 36 | 471 | 39 | 262 | 22 | 99 | 8 |
| **P-value** |  |  | 0.7993 | | **0.0114** | | 0.4467 | | 0.7627 | |
| **Rural vs urban residence** | | | |  |  |  |  |  |  |  |
| Urban | 704 | 30 | 271 | 38 | 289 | 41 | 166 | 24 | 64 | 9 |
| Rural | 1646 | 70 | 560 | 34 | 543 | 33 | 368 | 22 | 135 | 8 |
| **P-value** |  |  | 0.2097 | | **0.0242** | | 0.6377 | | 0.6141 | |
| **Chronically malnourished^5^** |  |  |  |  |  |  |  |  |  |  |
| Yes | 1122 | 48 | 427 | 38 | 408 | 36 | 235 | 21 | 109 | 10 |
| No | 1228 | 52 | 404 | 33 | 423 | 34 | 300 | 24 | 90 | 7 |
| **P-value** |  |  | 0.0545 | | 0.4685 | | 0.1372 | | 0.5559 | |
| **Children < 5 years in household** |  |  |  |  |  |  |  |  |  |  |
| < 3 children | 1602 | 68 | 557 | 35 | 552 | 34 | 356 | 22 | 127 | 8 |
| ≥ 3 children | 747 | 32 | 274 | 37 | 280 | 37 | 179 | 24 | 73 | 10 |
| **P-value** |  |  | 0.4799 | | 0.3468 | | 0.5447 | | 0.2963 | |
| **Malaria (blood smear) result** |  |  |  |  |  |  |  |  |  |  |
| Negative | 1825 | 78 | 604 | 33 | 667 | 37 | 422 | 23 | 156 | 9 |
| Positive | 525 | 22 | 226 | 43 | 165 | 31 | 112 | 21 | 43 | 8 |
| **P-value** |  |  | **0.0018** | | 0.1113 | | 0.4659 | | 0.8625 | |
| **Total** | **2350** | **100** | **525** | **22** | **525** | **22** | **534** | **23** | **200** | **9** |
| ^1^Percentage is out of total children. | | | | | | | | | | |
| ^2^Percentages for fever, cough, diarrhea, and fever + cough + diarrhea indicate per cent out of variable category. | | | | | | | | | | |
| ^3^Wald chi-square test for independence of column and row variables. | | | | | | | | | | |
| ^4^Wealth index is the Demographic and Health Survey composite measure of a household's cumulative living standard. Based on household ownership of previously selected assets, and utilizing principal components analysis, households are placed within one of five quintiles. The dichotomized variable has combined the two lowest categories into the "Poor" category and the three wealthiest into the "Middle income/wealthy" category. | | | | | | | | | | |
|  |  |  |  |  |  |  |  |  |  |  |
| ^5^Calculated according to NCHS/CDC/WHO international reference standard for height/age, dichotomized as -2.0 to ≤ -3.0 standard deviation (SD) below the mean (chronically malnourished), and normal to ≥ 3.0 SD above the mean (normal/overnourished). | | | | | | | | | | |
|  |  |  |  |  |  |  |  |  |  |  |
