# Supplementary material for: Association of Previous Measles Infection With Markers of Acute Infectious Disease Among 9- to 59-Month-Old Children in the Democratic Republic of the Congo
Source: J Pediatric Infect Dis Soc. 2018 Oct 19;8(6):531–8. doi: 10.1093/jpids/piy099 (PMC6933309; doi:10.1093/jpids/piy099)
Supplement: piy099_suppl_Supplementary_Table_3 [file piy099_suppl_supplementary_table_3.docx]

| Supplementary Table 3: Sensitivity analysis examining association of measles disease history with acute infectious disease episode of fever, cough, diarrhea, and fever + cough + diarrhea, considering varying levels of maternal report accuracy. | | | | | | |
| --- | --- | --- | --- | --- | --- | --- |
|  |  |  |  |  |  |  |
|  |  |  | **Fever** | **Cough** | **Diarrhea** | **Fever + Cough + Diarrhea^3^** |
| **Model** | **Measles status^2^** | | **OR and 95% CI^1^** | **OR and 95% CI** | **OR and 95% CI** | **OR and 95% CI** |
|  |  |  |  |  |  |  |
|  | **Classified + (n)** | **Classified - (n)** |  |  |  |  |
| **One (29% of cases)^4^** | 51 | 2298 | 1.49 (0.85, 2.61) | 1.07 (0.55, 2.08) | 0.78 (0.37, 1.61) | 1.44 (0.61, 3.37) |
| **Two (39% of cases)** | 74 | 2276 | 1.36 (0.78, 2.38) | 1.02 (0.53, 1.94) | 0.82 (0.41, 1.66) | 1.37 (0.56, 3.36) |
| **Three (49% of cases)** | 98 | 2252 | **1.80 (1.11, 2.93)** | 1.48 (0.87, 2.52) | 1.45 (0.72, 2.92) | 2.26 (0.99, 5.17) |
| **Four (59% of cases)** | 115 | 2235 | **1.69 (1.09, 2.62)** | 1.20 (0.72, 2.02) | 1.13 (0.66, 1.93) | 1.57 (0.65, 3.77) |
| **Original (100% of cases)** | 193 | 2157 | **1.80 (1.25, 2.60)** | 1.24 (0.82, 1.86) | 1.24 (0.80, 1.93) | 1.74 (0.96, 3.15) |
| ^1^Controlling for the following additional covariates: measles vaccination (vx), wealth index (WI), vx*WI interaction, breastfeeding, maternal education, parity, age, sex, malaria positive status, rural versus urban residence, residence*WI interaction, (old) province, vx*province interaction, and chronic malnutrition (according to NCHS/CDC/WHO international references standard for height/age SD). | | | | | | |
|  |  |  |  |  |  |  |
| ^2^2350 observations used in the regression model for all outcomes of fever, cough, diarrhea, and fever+cough+diarrhea. | | | | | | |
| ^3^Due to reduced number of outcomes for fever + cough + diarrhea, the vx*province interaction variable was removed from the model. | | | | | | |
| ^4^Percentage indicates measles reports incorporated in the model as measles positive out of total measles reports. Measles reports not included in a given model were considered to be measles negative. | | | | | | |
