# Supplementary material for: Association of Previous Measles Infection With Markers of Acute Infectious Disease Among 9- to 59-Month-Old Children in the Democratic Republic of the Congo
Source: J Pediatric Infect Dis Soc. 2018 Oct 19;8(6):531–8. doi: 10.1093/jpids/piy099 (PMC6933309; doi:10.1093/jpids/piy099)
Supplement: piy099_suppl_Supplementary_Table_4 [file piy099_suppl_supplementary_table_4.docx]

| Supplementary Table 4: Frequency data of acute infectious disease episode of fever, cough, or diarrhea in the previous two weeks among children 9-59 months of age by months since measles disease. | | | | | | | |
| --- | --- | --- | --- | --- | --- | --- | --- |
|  |  |  |  |  |  |  |  |
|  | **Category Total** | **Fever** | | **Cough** | | **Diarrhea** | |
| **Time since measles (months)** | **n** | **n** | **(%)** | **n** | **(%)** | **n** | (%) |
| **Model 1 frequency data** |  |  |  |  |  |  |  |
| No measles history | 2157 | 746 | 35 | 759 | 35 | 492 | 23 |
| 2-12 months since measles | 62 | 31 | 50 | 29 | 47 | 16 | 26 |
| 12-23 months since measles | 54 | 26 | 48 | 16 | 30 | 14 | 26 |
| 24-57 months since measles | 60 | 24 | 40 | 23 | 38 | 11 | 18 |
| **Model 2 frequency data** |  |  |  |  |  |  |  |
| No measles history | 2157 | 746 | 35 | 759 | 35 | 492 | 23 |
| 2-9 months since measles | 51 | 24 | 47 | 26 | 51 | 14 | 27 |
| 10-18 months since measles | 49 | 23 | 47 | 15 | 31 | 14 | 29 |
| 19-27 months since measles | 30 | 16 | 53 | 13 | 43 | 4 | 13 |
| 28-36 months since measles | 24 | 11 | 46 | 10 | 42 | 6 | 25 |
